# Anterior Cruciate Ligament Arthroscopic Ligamentoplasty Using the Four-Strand Hamstring Technique

**DOI:** 10.7759/cureus.40330

**Published:** 2023-06-12

**Authors:** Amer Yahia

**Affiliations:** 1 Emergency Department, Cheikh Khalifa Bin Zayed Al Nahyan Hospital, Casablanca, MAR

**Keywords:** tibial tunnel, femoral tunnel, ligament reconstruction, arthroscopy and sports related injuries, anterior cruciate ligament (acl) reconstruction

## Abstract

In this study, we looked at the clinical outcomes of individuals who had a symptomatic torn anterior cruciate ligament and had their anterior cruciate ligament repaired using a four-strand hamstring (4SHS) tendon autograft at least two years later. This is a retrospective study of 34 cases of anterior cruciate ligament arthroscopic reconstruction using the four-strand hamstring graft, collected from Traumatology and Orthopedics at Cheikh Khalifa Hospital in Casablanca, Morocco between January 2017 and January 2021. Different surgeons performed the operations. Physical findings and functional scores were recorded before the follow-up physical examination and surgery; thus, knee radiographs have been evaluated. A six-month rehabilitation program was devised following surgery. The average age of the patients was 30 years, with a male predominance of 94%. Thirty patients (88%) reported negative pivot shift tests and the Lachman test. The average Lysholm score enhanced from 59.3 before surgery to 85.29 at the time of the assessment. Three patients (8.82%) with a positive pivot shift test had no background of extra knee damage. In comparison to the appearance on the preoperative radiographs, no proof of progressive degenerative deterioration was found on the follow-up radiographs. All of the patients, however, had tunnel extension. The tibial tunnel grew on average by 18%, while the femoral tunnel grew on average by 30%. In 88% of patients assessed at least two years after surgery, ligamentoplasty of the anterior cruciate ligament using the four-strand hamstring graft eradicated anterior tibial subluxation. The failure rate was overall 10%. The functional knee scores had greatly improved at the time of the follow-up.

## Introduction

The four-strand hamstring (4SHS) ligamentoplasty of the anterior cruciate ligament (ACL) efficiently removes anterior tibial subluxation caused by ligament rupture.

To restore the injured native ligament, current anterior cruciate restoration techniques use a range of autograft and allograft tissue. An excellent method of curing anterior tibial subluxation caused by a torn of anterior cruciate ligament is reconstruction with an autologous bone-patellar tendon-bone graft. Despite the fact that this graft type remains the "gold standard" for reconstructing the anterior cruciate ligament, the potential morbidity of this procedure (patellofemoral pain, loss of motion, and patellar fracture) has prompted further research into the use of alternative graft sources that would yield clinical results comparable to those seen after reconstruction with a bone-patellar tendon-bone graft [1].

Anterior cruciate ligament reconstruction with a hamstring (semitendinosus and gracilis) tendon autograft has previously been documented. At time zero and with cyclical loading, the four-strand semitendinosus and gracilis construct was found to be equal to or greater than the strength of a bone-patellar tendon-bone graft of identical dimension. Although studies of four-strand constructs have recently been published, most published publications on ACL reconstruction with autogenous hamstring tendon grafts have detailed clinical results in individuals treated with a two- or three-tendon graft [2].

We expected that using a four-strand hamstring tendon autograft in an arthroscopically assisted anterior cruciate ligament reconstruction would reduce evidence of anterior tibial subluxation and offer a favorable functional result for patients with a damaged ACL. Thus, we drove a clinical trial to assess the efficiency of a four-strand, double-looped, semitendinosus and gracilis autograft used in a double-incision, arthroscopically assisted restoration of the ACL.

## Materials and methods

Patients and entry criteria

Our institutional review board gave their approval to this retrospective study's protocol. Following that, all patients suffering from a ruptured ACL with a symptomatic anterior tibial subluxation completed a standardized evaluation which included a physical examination (the pivot shift test, Lachman test, and measurement of range motion) and took anteroposterior and lateral radiographs of the knee over a two-year period, with symptomatic anterior tibial. At the presentation, the Lysholm and Tegner knee function scores were acquired.

Patients from our study received a four-strand semitendinosus and gracilis tendon autograft through a double-incision, arthroscopically assisted procedure. Thirty-four patients were treated in this manner in a row. During the study period, the operating surgeons did not execute any other type of reconstruction for isolated symptomatic anterior cruciate ligament rupture. The average age of the patients at the time of surgery was thirty years (from seventeen to fifty years). Thirty-two male patients and two female patients were present.

The study excluded individuals who had an ACL tear associated with a posterior cruciate ligament tear, as well as patients who have already undergone homolateral ACL ligamentoplasty and patients who have undergone Kenneth-Jones-type ligamentoplasty.

Preoperative physical examination

**Figure 1:**
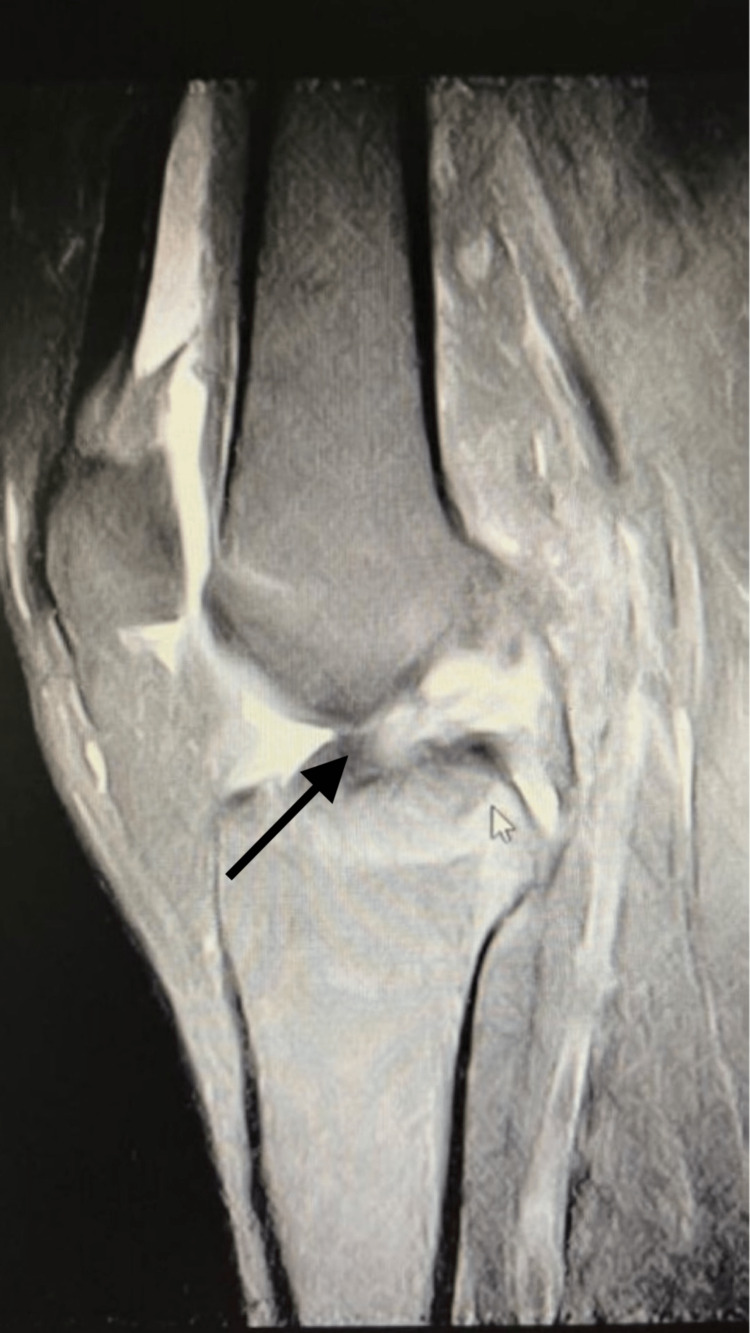
Preoperative MRI illustrating a complete rupture of the anterior cruciate ligament

The average knee flexion was 120° (from 100° to 140°), whereas the average knee extension was 0° prior to anterior cruciate ligament replacement. The Lachman test (grade 2) and pivot shift test (grade 1+) were both positive in all of the patients. The relative tibial displacement at 30° of flexion was graded by the Lachman test, where grade 1 refers to 0 up to 5 mm, grade 2 from 6 up to 10 mm, and grade 3 more than 10 mm. The Lachman test yielded a grade 1 result in no patient, a grade 2 result in twenty-two patients, and a grade 3 result in twelve patients. The degree of tibial reduction throughout the maneuver was used to grade pivot shift, where normal indicates no shift, 1+ (slight), 2+ (definite subluxation), and 3+ (subluxation and momentary locking). All the patients had an MRI which illustrated an anterior cruciate ligament rupture prior to surgery, as shown in Figure 1.

Surgical technique

**Figure 2:**
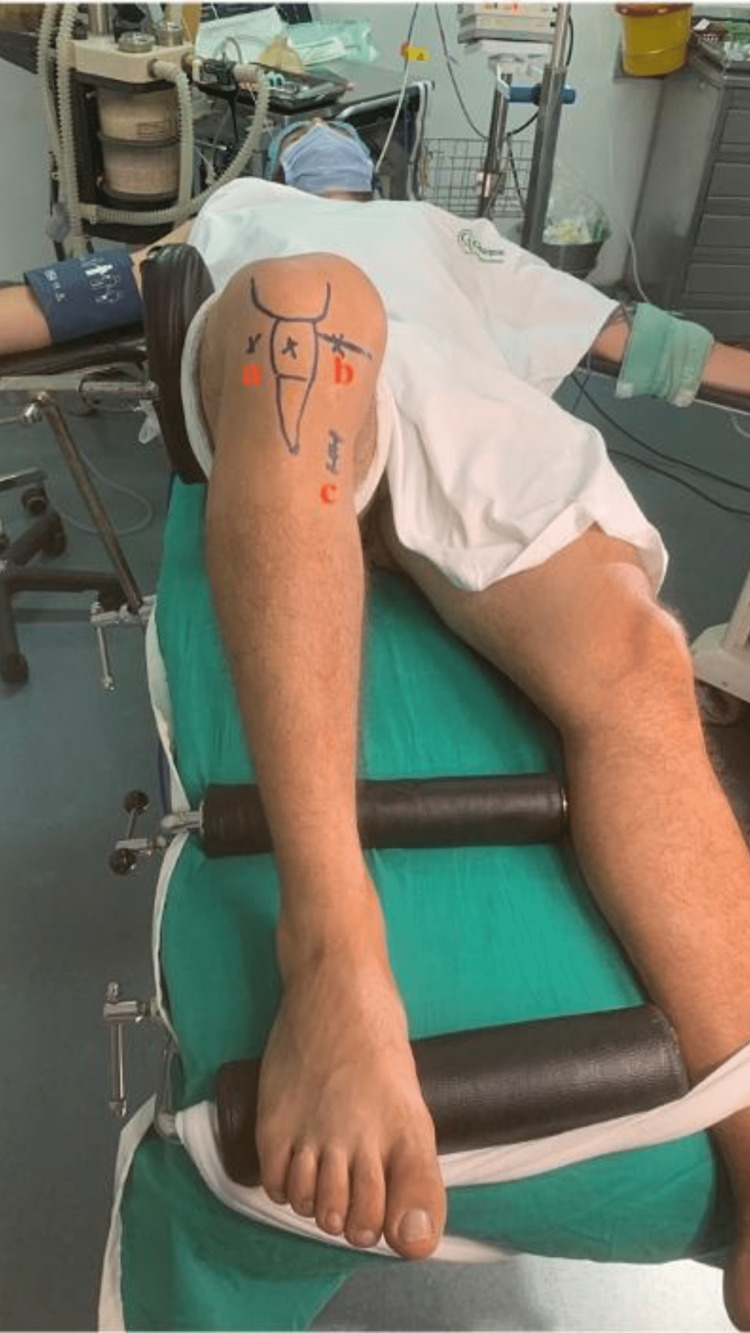
Surgical approaches a - anterolateral: high parapatellar b - antero-medial: lower parapatellar c - identification of crow's feet

In Figure 2, surgical approaches are displayed. A double-incision (high-parapatellar and lower para-patellar) arthroscopically aided anterior cruciate ligament reconstruction was performed. Prior to the skin incision, antibiotics were given. The limb to be operated on was first cleansed. Distal to the tibial tubercle, an adhesive plastic barrier drape was covering the leg. As previously mentioned, the semitendinosus and gracilis tendons were extracted from the ipsilateral limb. In all of the surgeries, a tendon harvester was used. To prepare the graft, all muscle fibers were removed from the retrieved tendons.

Both tendons were threaded over a closed-loop polyester tape attached to an interference screw, resulting in four tendon strands, each with a free end. A non-absorbable braided number 1 suture was used to tether each free tendon end in an interlocking method. 

Diagnostic arthroscopy was conducted after the hamstring transplant was prepared. Prior to the anterior cruciate surgery, any meniscal damage was treated. A lateral femoral notchplasty was done on each patient to improve the visibility of the posterolateral intracondylar region of the femoral condyle. The diameter of the graft from each patient was used to generate the tibial and femoral tunnels. After the femoral tunnel was created, between the anterior femoral cortex and the proximal section of the femoral tunnel, a 4 mm tunnel was formed.

The interference screw suture-loop complex was established after a depth assessment to help with the femoral fixation of the hamstring transplant. Within the femoral tunnel, a minimum of 25 mm of tendon graft was inserted in each patient.

After securing the interference screw to the femoral cortex's exterior, while tension was given to the graft, the knee was flexed and extended. Before tibial fixation was applied, individual graft limbs were individually tensioned.

Bioabsorbable interference screws (34 patients) were used in both femoral and as well as for tibial fixation, which was done with the knee at 20° of flexion.

Associated procedures at index operations

Three patients had partial lateral meniscectomy, and six patients had partial medial meniscectomy at the time of anterior cruciate repair. Ten patients had a meniscal repair where eight had a medial meniscal repair, and two had lateral meniscal repair using the Acufex meniscal suture repair system.

Postoperative rehabilitation system

All patients underwent a six-month rehabilitation program, identical to one previously described for anterior cruciate repair. They directly began passive range-of-motion exercises and active quadriceps isometrics. There were no continuous-passive-motion devices deployed. Patients who underwent surgery in 2017 were permitted to bear weight on the surgically treated leg immediately following surgery, putting the knee in a hinged brace that was fully extended. Patients used a brace postoperatively in the second year of the research but were only allowed to toe-touch weight-bearing during the first three weeks following surgery on crutches. In both groups of patients, the remainder of the supervised rehabilitation approach was the same.

Postoperative assessment

At the follow-up time, all exams were performed by a single examiner. The physical examination included measuring the knee's range of motion with a portable goniometer, Lachman testing, and pivot shift testing. All patients filled out questionnaires to determine their Lysholm and Tegner ratings, and all underwent standing anteroposterior and lateral radiographs of the surgically repaired knee.

Statistical methods

Using the patients' medical files, surgical reports, and consultation records, all data was gathered on a data sheet. The Lysholm and Tegner score sheet was used to evaluate functional outcomes. Microsoft Office Excel was used for data input.

Objectives

The aim of this study is to evaluate the clinical outcomes of ACL tear surgery utilizing the 4SHS approach, as well as compare our findings to data from the literature.

## Results

Patient follow-up

At a mean of twenty-eight months, 34 patients (32 males and two females) were available for a complete physical examination. Functional ratings were acquired using a questionnaire at the follow-up exam.

Clinical assessment

Seven (20.5%) of the thirty-four patients had flexion contractures of less than 5° at the follow-up exam. The average flexion was 135°, and the average extension was 0.2°. Nine (26.47%) of the 34 patients had a positive (grade 2) Lachman test, including six who had a significant rip of the hamstring graft following surgery.

At the time of follow-up, nine (26.47%) of the thirty-four patients had a positive pivot shift test (1+ or 2+). Six of them experienced a recurrence of knee instability following a particular injury to the surgically repaired knee.

The use of bioabsorbable screws had no effect on the findings of the Lachman and pivot shift tests.

Functional scores

In our study, 89.5% of patients were classified as "good" or "excellent" on the Tegner scale. Ligamentoplasty allowed these patients to resume a normal lifestyle and sports activities, perform unrestricted knee motions without locking, squat beyond 90°, and ascend and descend stairs without difficulty.

These individuals now enjoy a painless and stable knee. Furthermore, five patients (14.70%) were unsatisfied - the "average" category - mostly due to discomfort, limitation of knee motions while squatting, and difficulty climbing stairs. In Table 1, according to the Lysholm and Tegner scale, the mean global score increased from preoperative 59.3 to postoperative 85.29.

**Table 1:** Results of the average Lysholm and Tegner pre- and postoperative score

Results	Lysholm and Tegner score
Preoperative	59.3
Postoperative	89.5

Rehabilitation protocols

With supporting permission, we followed the identical rehabilitation plan for all of our patients 24 hours following surgery. The physiotherapy staff at Cheikh Khalifa Bin Zayed Al Nahyan Hospital provided this program during our patients' stay. Patients were allocated to outpatient rehabilitation after being discharged from the hospital. It should be mentioned that due to its inaccessibility, rehabilitation was not always acknowledged by our patients. Our patients' recovery lasted an average of six weeks, with an average of 20 sessions.

Radiographic assessment

**Figure 3:**
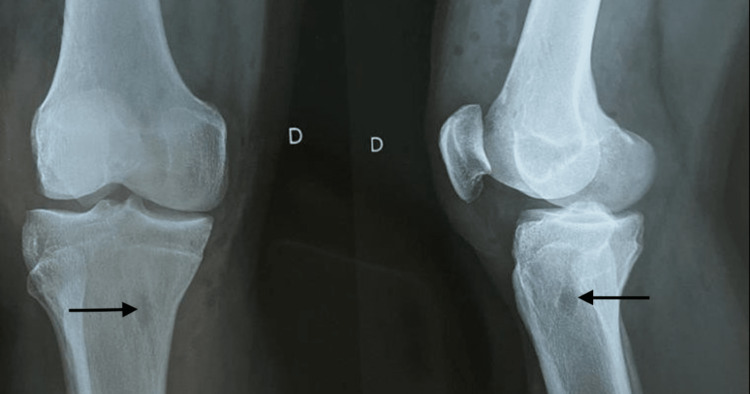
X-ray of the knee showing the tibial and femoral fixation using a screw

The pretreatment and follow-up knee radiographs were evaluated by one radiologist. The femoral and tibial tunnels created during the anterior cruciate reconstruction were measured on anteroposterior and lateral radiographs taken at the first postoperative visit, seven to ten days after surgery, as shown in Figure 3, and the measurements were compared to those on knee radiographs taken at the follow-up examination. The tunnel expansion over the follow-up interval was calculated using the maximum width of each tunnel. The use of radiographic magnification was contemplated. The rate increase in tunnel width on follow-up radiographs was 14% on the anteroposterior radiograph and 18% on the lateral radiograph as compared to the width on immediate postoperative radiographs. Femoral tunnel enlargement averaged 30% on the anteroposterior radiograph and 8% on the lateral radiograph. Negative tunnel expansion findings are known to imply tunnel shrinkage. In our study, the tunnel size was less at follow-up than it was immediately after surgery. There was a larger degree of tibial tunnel expansion on the lateral radiographs and a bigger degree of femoral tunnel expansion on the anteroposterior radiographs. The use of bioabsorbable screw fixation had no effect on femoral or tibial tunnel width.

Postoperative complications

There were no intraoperative incidents or conversions to open surgery recorded in our series. Our patients experienced no acute postoperative problems, including no cases of surgical site infection, thromboembolic complication, or osteoarthritis.

## Discussion

The treatment of anterior cruciate ligament ruptures has advanced significantly over time, owing mostly to the discovery of novel surgical procedures and the improvement of these techniques under arthroscopy, allowing for better placement of the graft and more successful treatment of associated meniscal and cartilaginous lesions. 

According to Frank and Jackson [3] and Grøntvedt et al. [4], ACL reconstruction using tendon autograft lowers laxity, corrects knee instability, and restores a higher level of activity compared to initial repairs with tendon suture, which has a failure rate of 100%.

Many grafts can be used: the patellar tendon, the fascia lata, and the hamstring tendons, which respond to several surgical techniques. For example, the Kenneth-Jones (KJ) technique uses the patellar tendon, the 4SHS technique uses two tendons of the internal rectus and the semitendinosus, the demi tendinosis 4 grafts tape locking screw (DT4 TLS) technique uses the semitendinosus tendon and the Macintosh fascia lata (FL) technique uses a strip of fascia lata.

Because of the differences in reported surgical procedures, it is difficult to compare the outcomes of previous investigations. Aglietti [5] recommends using his method of radiological tunnel location measurement to precisely correlate the position of the tunnels with a failure of stability. In his research, 62.5% of patients with an anterior femoral tunnel (50% of the femoral condyle) had inadequate anterior instability, compared to 12% of patients with an aperture in the posterior half of the condyle. On the other hand, locating the tibial tunnel too anteriorly (20% of the tibial plateau) worsens the knee's extension deficit - an opening situated in the anterior 15% greatly enhances this loss of extension by at least 5°. 

The accuracy with which the tunnels are positioned assures the success of ACL surgery. Aglietti [5] claims that the best outcomes are produced when the reconstruction is essentially anatomical. A tibial tunnel positioned too anteriorly causes a conflict between the graft and the intercondylar notch, resulting in an extension deficit, anterior discomfort with residual effusion, instability, and eventually graft rupture. Similarly, an anterior femoral tunnel will induce greater graft elongation during knee flexion and will cause the graft to burst rather fast.

The length of the graft in our study was 7 cm, which corresponds to the total length of the intra-articular route and the depth of the two cells. Research [6] published in the literature examined the length of the grafts in two groups of patients operated on using two surgical techniques: 15 patients with a full ACL rupture operated on using the tape locking screw® (TLS) method, and 15 patients operated on using the 4SHS technique. There were no intraoperative or postoperative problems in either group; the mean graft length in the 4SHS group was 7.4 cm and 9.2 cm in the TLS group.

For the graft fixation, Katabi and his colleagues [7], particularly in the 4SHS group, discovered that femoral fixation by EndoButton offers excellent resistance to rupture but poses a problem due to the reduction in rigidity of this assembly, resulting in knee laxity, whereas the use of an interference screw allows for a more rigid anatomical fixation with significant knee stabilization. Ishibashi et al. [8], Guirea et al. [9], and Weiler et al. [10] all reported the same finding. Nonetheless, at the tibial level, the quality of the screw fixation was unsatisfactory, necessitating the inclusion of a second means of fixation, particularly a staple, in 25% of the instances.

Concerning our study, 34 patients were available for a complete physical examination after an average of twenty-eight months. At the follow-up exam, functional evaluations were obtained using a questionnaire. At the same time, other studies exposed that the mean follow-up was much longer. For instance, Niu et al. [11], in their study, demonstrated that the mean follow-up was forty months. 

Following the ACL reconstruction, residual patellofemoral discomfort has been documented in various investigations, independent of the procedure utilized. Residual pain was observed in just 6.44% of our patients, which is comparable with studies by Bedin [12], Marder et al. [13], and Aune et al. [14]. According to the authors, the 4SHS group experienced much less discomfort than the KJ group. In our study, two patients (5.88%) experienced knee instability, which is similar to the findings of the Bedin (4.8%) [12] and Mossaid (5.56%) series [15]. There was no evidence of instability in the Katabi et al. series [7].

Concerning knee examination and particularly joint amplitude, knee flexion stiffness was noted in 5.56% of our patients, which is similar to the Mossaid series (16.7%) [15]. In contrast, Katabi et al. [7] did not include flexion stiffness in their series. The stiffness in extension (flessum) recorded in our series (1.85%) is likewise reported in 2.25% of instances by Katabi [7] and 5.56% of cases by Mossaid [15]. The Lachman test, anterior drawer, and leap sign were all negative in all of our patients, which was similar to the Mossaid series [15]. Jardin et al. [16] questioned the accuracy of the KT-1000 arthrometer in measuring residual laxity. 

In our everyday practice, the only technique to quantify ligament laxity is through clinical examination, which renders our results subjective. According to the literature, residual laxity is more pronounced in the 4SHS method than in the KJ approach. The 4SHS and KJ groups had residual laxity of 4.5 mm and 2.7 mm in Katabi [7], 4 mm vs. 3.4 mm in Aglietti [5], 2.8 mm and 2.3 mm in Maletis et al. [17], and 2.5 mm and 1 mm in Bedin [12].

The Lysholm and Tegner scale is the most extensively used at the moment. Since it was originally designed for the follow-up of ACL ligament repairs, its clinical usefulness and sensitivity have yet to be determined [18]. This scale is worth a total of 100 points. The functional portion of the questionnaire questions the patient about lameness (5 points), walking help (cane; 5 points), squatting (5 points), stair climbing (10 points), instability (25 points), and obstruction (15 points). This functional evaluation is completed by clinical criteria of effusion (10 points) and discomfort (25 points). A score of 83 or more is regarded as good or very good, 65-83 as ordinary, and 65 or lower as bad. Based on an examination of 11 studies, Choary and Poiraudeau conclude that this scale is replicable and valid [19]. In 2015, the Société Française d'arthroscopie released an essay on the long-term prospects of anterior cruciate ligament ligamentoplasty [20]. The Lysholm score was used to evaluate ligament repairs after surgery. 

The analysis of the results, which show the average Lysholm score after ACL ligamentoplasty according to different methods reported by several authors, leads us to the conclusion that the 4SHS technique had a slightly higher score than the other techniques, such as KJ or Macintosh FL.

## Conclusions

The anterior cruciate ligament is important in knee kinematics because it stabilizes the knee across a large range of motion. As proven by many authors, an ACL lesion causes knee instability, which prevents a return to prior activity and modifies kinematics owing to the adaptation of the other structures of the knee to compensate for the ACL deficiency. Despite this adaptability, which is mostly due to musculature, this shift in movement can lead to degenerative meniscal and cartilage lesions over time, encouraging surgical reconstruction. Several surgical procedures for ACL restoration have been published in the literature; two of the most commonly used are the 4SHS approach and the KJ technique. The strong points of the 4SHS technique used in our training are, on the one hand, the use of a solid transplant, which provides a very satisfactory fixation, and, on the other hand, the respect for the extensor apparatus, which allows for early rehabilitation and thus avoids the frequent morbidity problems associated with transplant removal (KJ technique). Nonetheless, the 4SHS group has less residual discomfort. According to the clinical examination and the functional score of Lysholm and Tegner, the overall outcomes in our series were good in terms of stability and postoperative morbidity. The results show that using this approach not only allows for the management of laxity but also has a low morbidity rate. However, our follow-up duration is restricted, and research with a longer follow-up period and a bigger team are required.
